# Turning the World Upside Down to Understand Perceived Transparency

**DOI:** 10.1177/2041669516671566

**Published:** 2016-09-26

**Authors:** Juno Kim, Phillip J. Marlow

**Affiliations:** School of Optometry and Vision Science, University of New South Wales, NSW, Australia; School of Psychology, University of Sydney, NSW, Australia

**Keywords:** vision, illumination, material perception, refraction, transparency, glass, gloss

## Abstract

Specular surfaces and refractive media are difficult to distinguish from each other because they both generate distorted images of the surrounding lighting environment. Whereas convex refractive objects invert the orientation of the horizon so the sky appears beneath the ground plane, convex specular surfaces preserve the orientation of the horizon so the sky appears above the ground. Here, we show that a refractive transparent object can be made to appear specular and opaque simply by rotating the image by 180°. This result suggests that the visual system relies on information tied to the orientation of the horizon to distinguish between refractive and specular objects.

Figure 1 compares a glass sphere (a) and a perfect mirror sphere (b) embedded in the same light field. The most salient difference between the two materials is the orientation of the horizon, which is right side up for the mirror sphere, but upside down for the glass sphere. The orientation of the horizon differs between the glass and mirror spheres because refraction and specular reflection project light in different directions relative to the surface normal (Figure 1, lower). Specularly reflected light has a similar direction to the outward facing surface normal, which bisects the angle between the incident and specularly reflected rays. Refracted light, on the other hand, is bent toward the inward facing surface normal when it enters glass and is then bent away from the outward facing surface normal when returning to air.

**Figure 1.**
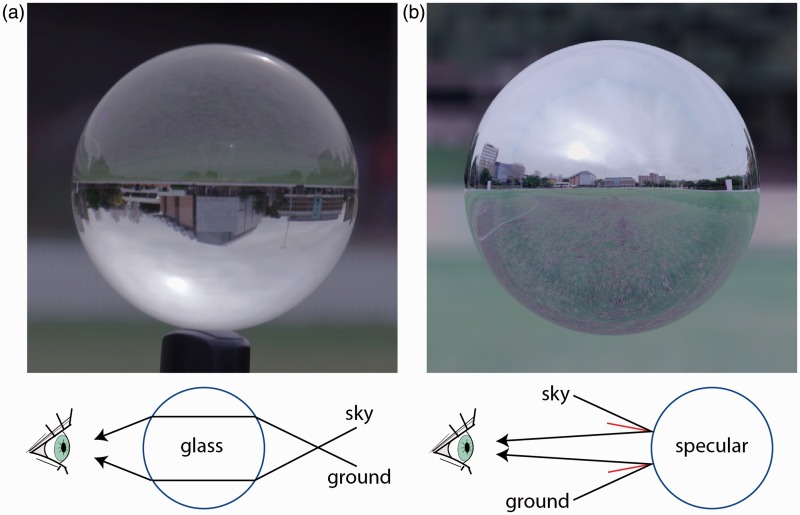
Distortions of the light field generated by refractive (a) and reflective (b) convex objects. (a) Photograph of a real refractive glass ball (10 cm diameter) at 2 m in an outdoor lighting environment (Village Green Oval, UNSW, Sydney, Australia). (b) A comparable purely specular version of the same 3D shape simulated in the same illumination environment. The HDR specular light probe of the UNSW Oval was simulated in Blender 3D from three auto-exposure bracketed images on a Canon 5D Mark III with 180° fisheye lens (see Debevec, 2002). Schematics illustrate the transport of light rays from the sky and ground for refraction and reflectance below each image.

We tested whether the orientation of the horizon functions as a cue for distinguishing between refractive media and reflective surfaces. Observers were presented with upright (Figure 2(a)) and 180° rotated (Figure 2(b)) images of a perturbed spherical geometry rendered as refractive media using the physically based rendering software Maxwell Render (∼10 cm diameter with an index of refraction of 1.5111 and no roughness). The simulation parameters were based on measured data for K7 Crown glass preconfigured in the rendering software. Relief perturbation was generated by displacing vertices in the radial direction by distances determined by a cloud noise texture. The camera was situated 30.5 cm from the center of the object. We observe that the upright image (Figure 2(a)) generates a compelling percept of transparency (as though it is constructed entirely from ice or glass), whereas the inverted image (Figure 2(b)) is misperceived as more opaque and reflective.

**Figure 2.**
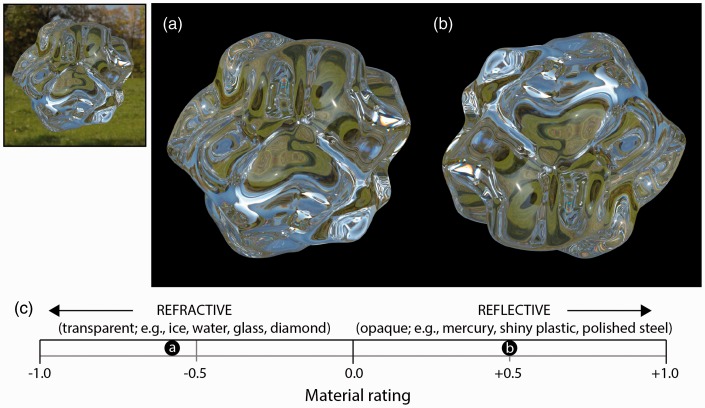
An image of a transparent bumpy 3D object rendered with a refractive index of 1.5111 (a) and the same image rotated by 180° (b). The inset shows the glassy object rendered with the outdoor light field visible in the background as generated in Maxwell Render V3 (50 mm focal length, 1/60 s shutter speed, and 32 f-stops). Mean confidence judgments for objects in the two images appearing refractive versus reflective are shown in (c).

We measured this image inversion effect by instructing eight naïve observers to rate the strength of perceived transparency or opacity when the image was presented in upright and inverted orientations. The order of presentations was counterbalanced across observers. Ratings were ordered metrically along a continuum from −1.0 through 0 to +1.0. The extremity of the rating from zero conformed to the observer’s confidence in their rating of one material classification over the other (zero meaning complete ambiguity).

Figure 2(c) shows mean ratings across all observers for the upright and inverted images. The upright refractive object was judged as transparent and reflective with a mean rating of −0.58 (*SE* = 0.12), whereas the surface in the inverted image was judged as opaque and reflective with a mean rating of +0.50 (*SE* = 0.05). Ratings were significantly different from zero-level uncertainty for the object depicted in both the upright image (*t*_7_ = 4.99, *p* < .005) and inverted image (*t*_7_ = 9.50, *p* < .0001). These results confirm an image inversion effect, supporting the view that perceived transparency depends on the inferred orientation of the horizon in the light field, assumed to be structured with prevailing illumination from above.

All observers indicated after their participation that the objects appeared globally convex. This is important because concave specular surfaces will also generate an inverted image of the horizon. For example, the convex specular sphere in Figure 1(b) can appear concave if its image is viewed upside down. This observation suggests that perceived convexity and perceived reflectance codepend on one another. Therefore, the visual system would need to account for perceived convexity of the object to derive surface reflectance from the apparent orientation of the horizon.

Another distinguishing feature of refractive objects is the *distortion field*—patterns of magnification and minification in the refracted structure of light. For a fixed 3D shape and light field, increasing refractive index increases the amplitude of the distortion field and increases ratings of the perceived refractive index (Fleming, Jäkel, & Maloney, 2011; Ueda et al., 2015; c.f., Schlüter & Faul, 2014). However, increasing refractive index also reduces the critical angle for total internal reflection. Total internal reflection generates significant banding of image structure at regions of high curvature (Figure 2(a)), which may provide additional information about the refractive properties of thick transparent objects.

The image-inversion effect we report here adds to previous literature by suggesting that the global orientation of photometric variations in these distortions, and not just the local structure of distortions per se, contributes to perceived transparency. This finding compliments other studies that have identified illumination biases in the perception of other surface properties, including 3D shape (e.g., Mamassian & Goutcher, 2001) and pigmentation (e.g., Kim, Marlow & Anderson, 2014).

One potential caveat is that the image inversion cue requires a strongly directional, anisotropic light field. Our results suggest it would be fruitful to further explore how the structure of the light field influences observer judgments of perceived shape, opacity, and refractive index.
